# Comorbidity among HHT patients and their controls in a 20 years follow-up period

**DOI:** 10.1186/s13023-018-0962-8

**Published:** 2018-12-14

**Authors:** Katrine Saldern Aagaard, Anette Drøhse Kjeldsen, Pernille Mathiesen Tørring, Anders Green

**Affiliations:** 1 Danish HHT Center OUH, Department of Otorhinolaryngology, Odense University Hospital, 5000 Odense C, Denmark; 2 Clinical institute University of Southern Denmark, Odense, Denmark; 3 Department of Clinical Genetics, Odense University Hospital, Odense, Denmark; 4 Odense Patient data Explorative Network (OPEN), Odense University Hospital/Department of Clinical Research, University of Southern Denmark, Odense, Denmark

**Keywords:** Hereditary haemorrhagic telangiectasia, HHT, Infections, Comorbidity, Hospitalisation

## Abstract

**Background:**

Hereditary Haemorrhagic Telangiectasia (HHT) is an autosomal dominant genetic disorder with a wide variety of clinical manifestations due to the presence of multiple arteriovenous malformations in various tissues and organs.

**Objective:**

To study the need for hospital admittance in a group of HHT patients and matched controls during a 20 years follow-up period commencing in 1995.

**Methods:**

All HHT patients in the County of Funen, Denmark, were included. For each patient, three age and sex matched controls were identified at the time of enrolment. Data on all hospitalisations were extracted from the national health registers and compared with clinical records. The hospitalisations were grouped as HHT relevant or not HHT relevant based on the discharge diagnosis (International Classification of Diseases, ICD10) and with particular focus on infections, bleedings and thromboembolic events.

Patients with HHT were compared with controls concerning the first time incidence of each discharge diagnosis.

**Results:**

We included 73 HHT patients and 219 controls of which one control was lost to follow-up. HHT-patients had significantly more hospitalisations per person caused by infections in joints and bones, but not caused by infections in general. Bleeding episodes were, as expected, more frequent among the HHT-patients. The study revealed a similar incidence of abscesses and thromboembolisms, including in the central nervous system, among the HHT patients and controls.

**Conclusions:**

Based on this study Danish HHT patients had an increased comorbidity of infections in joints and bones and of bleeding episodes. However, the incidence of thromboembolisms, cerebral abscesses and other conditions commonly considered related to HHT was comparable between the patients and the controls. The patients included in this study were closely monitored at a highly specialised HHT Centre where they received relevant diagnostic evaluation, treatment and counselling. Since this is assumed to benefit the overall health of the patients, it may explain why these patients were less prone to comorbidity than other studies have suggested.

**Electronic supplementary material:**

The online version of this article (10.1186/s13023-018-0962-8) contains supplementary material, which is available to authorized users.

## Introduction

Hereditary haemorrhagic telangiectasia (HHT), also known as Osler-Weber-Rendu disease, is a rare autosomal dominant disorder that gives rise to widespread arteriovenous malformations (AVMs.) The condition especially affects small superficial blood vessels in the skin and the mucosa and can also cause substantial AVMs in internal organs. The diagnosis is clinical and based on the Curaçao criteria [1]. In Denmark, the prevalence is about 1:6500 [2], which is similar to that of other populations [3]. The clinical presentation is highly variable: in some individuals, the symptoms are subtle and result in only minor inconvenience, others experience frequent heavy bleedings [2, 4]. Fatal outcomes have been reported occasionally [5, 6]. Some studies have found a reduced life expectancy among HHT patients [7–9]. However, in the present Danish HHT population mortality rates are not increased [10].

## Genetics of HHT

In most cases, HHT is caused by a mutation in one of two genes: *ENG* (MIM*131195) or *ACVRL1* (MIM*601284), giving rise to the two main subtypes: HHT1 and HHT2, respectively. A minor proportion of affected individuals have mutations in the *SMAD4* gene (MIM*600993), causing a combined syndrome of juvenile polyposis and HHT (JP-HHT) [11]. In around 90% of families with clinical HHT a disease-causing mutation is identified [12].

## Clinical manifestations of HHT

### Bleedings

The vast majority of affected individuals suffer from epistaxis and about half experience episodes beginning in childhood [13]. The severity and occurrence of bleeding episodes vary widely, as does the need for treatment and medical counselling [14].

Gastrointestinal bleedings are common among HHT patients (about 25%) and severe bleedings are more frequent in elderly patients. In some cases, the telangiectatic lesions can be identified by endoscopy and treated with laser therapy, however the success rate is often limited due to large numbers of widely scattered telangiectatic lesions [15].

### Arteriovenous malformations

A substantial part of HHT patients are affected by arteriovenous malformations (AVMs), which are found in multiple internal organs. Hepatic AVMs (HAVMs) are in screening studies observed in more than half of HHT patients, and most frequently in HHT2 patients [16, 17]. Nevertheless, severe symptomatic liver-disease is rare and is often related to high-output cardiac failure and portal hypertension [18]. Cerebral AVMs (CAVMs) are diagnosed in 11–23% of patients with HHT, most commonly among HHT1 patients [19, 20], and are associated with haemorrhage, migraine and seizures [21, 22]. About 35% of the HHT patients have pulmonary AVMs (PAVMs), occurring considerably more frequently in HHT1 patients [12, 16, 17, 23]. PAVMs can lead to dyspnoea [24], but often the patients present with asymptomatic hypoxemia [24, 25]. Even in the absence of symptoms, recognition and treatment of PAVMs is recommended [26, 27], as they compromise the filter function of the lungs, and therefore can be the cause of ischemic strokes and cerebral abscesses [23, 26–29]. PAVM haemorrhage leading to haemoptysis or haemothorax is a rare, but potentially life-threatening complication that also can be evaded by embolisation of PAVMs [30].

### Other HHT-related comorbidities

An association between HHT and severe extra-cerebral infections including septicaemia, arthritis, osteomyelitis, spondylodiscitis and endocarditis have previously been reported [31–35]. Prolonged epistaxis and nasal colonisation of *Staphylococcus aureus* may contribute to this association [32–35]. Moreover, elderly fragile patients affected by comorbidities are suggested to be more prone to these infections [35]. Several abnormalities of the adaptive immune system are associated with HHT [36]. Concurrently, iron deficiency, which HHT patients often experience in relation to frequent bleedings, is correlated with a reduced oxidative burst and impaired phagocytic activity [37]. It is not yet clear though, if these alterations impair the immune response.

The frequent bleeding episodes among HHT patients lead to an increased erythropoiesis, which can cause elevated blood viscosity. The combination of increased blood viscosity and iron deficiency, which is associated with pulmonary embolism/deep venous thromboses, suggests that HHT patients could be at increased risk of thromboembolic events [38]. Furthermore, paradoxical emboli passing through untreated PAVMs to the coronary arteries can lead to cardiac ischemia [39] and have in case reports been suggested as the cause of myocardial infarction [40, 41].

## Aim

The aim of this study was to evaluate the occurrence of thromboembolic and infectious diseases among HHT patients compared to matched controls. Furthermore, we wanted to assess the importance of bleeding episodes among HHT patients in terms of hospitalisations.

## Methods

The identification of HHT patients and controls has previously been described [42, 43]. Briefly, all known HHT patients and all first-degree relatives of HHT patients, living in the County of Funen, Denmark, were invited to a clinical examination of HHT signs at the Danish HHT Centre at Odense University Hospital, inclusion was based on outpatient clinical examination regardless of previous hospitalisation. A total of 73 patients fulfilled the clinical criteria of HHT on January 1st 1995 and were included in the study. The control group consisted of three age- and sex-matched controls for each HHT patient, in total 219 persons randomly chosen among all residents of the County of Funen as of January 1st 1995. Cases and controls were followed for 20 years with respect to hospital admissions and deaths. Results concerning mortality of this cohort have recently been reported [10].

### Clinical evaluation of patients

At the first visit at the HHT Centre, history taking and clinical examination focusing on HHT manifestations and neurological symptoms was performed and the patients were offered screening for PAVM. The screening included contrast echocardiography and if positive either a pulmonary angiography or a chest CT- scan was offered. If a PAVM with feeding arteries larger than 2 mm was identified, the patient was offered embolisation therapy [44]. Screening of CAVM and/or HAVM is not implemented as routine in Denmark. Screening for CAVM was offered if the patient had experienced neurological symptoms or deficits. In a few cases screening was performed in patients with concerns regarding CAVM. In all HHT families genetic counselling and mutation diagnostics were offered.

### Registry-based collection of follow-up data

At birth or upon immigration a unique civil registration number is assigned to all Danish residents. This number is used throughout in the private and public sector, including the Danish healthcare sector, for the registration of data related to a given individual. As part of the present study all relevant data on cases and controls were extracted from The Danish National Patient Register (Lands Patient Registeret, LPR). LPR is a nationwide register that since 1977 has registered information on all Danish residents hospitalised in somatic wards and from 1995 all hospitalisations and outpatient visits have been registered [45]. Data of relevance for the present study are specified in the section “Definition and grouping of relevant data” and a more detailed description can be found in Additional file 1: Appendix 1, online only. Our LPR data analysis included only hospitalisations with a date of discharge within the period from January 1st 1995 through December 31st 2014 or with an active contact as of January 1st 2015.

### Manual collection of follow up data

To validate and supplement the information acquired from LPR, all accessible medical data on patients and controls from the period January 1st 1995 through December 31st 2014 were obtained from the electronic administration-systems employed in the County of Funen during this period. The first data collection was performed in the period September–November 2014. For patients alive or recently deceased at the time of the first data-collection, a second review was performed in March 2015 to ensure inclusion of all data from the follow-up period. All hospital admissions and outpatient visits were individually reviewed to verify the consistency between the ICD-10 classification and the patient’s clinical presentation at each event, although only data regarding hospitalisation were employed for analysis, as stated above.

### Definition and grouping of relevant data

We applied up-to-date knowledge of HHT to the ICD10 classification, in order to categorise hospitalisations into “potentially HHT-related diagnoses” groups: infectious conditions, thromboembolic events, other vascular conditions and bleedings, including subgroups. (Table 1). All other hospitalisations were categorised as “non-HHT related conditions”. In the manually collected data, only HHT-related conditions were analysed. Here we also included all outpatient contacts involving treatment for epistaxis as these patients are often not hospitalised, and we evaluated and defined the group “Other HHT-related contacts”, which included other outpatient visits regarding HHT. In the manually collected data, secondary discharge diagnoses were included if the condition alone would have led to hospital admission. For elaborate description and classification of diagnoses, please see Additional file 1: Appendix 1, online only. To avoid bias due to individual patients with several hospitalisations of the same category, we decided to perform the analysis based only on the first registered event of a given category of contact for each individual patient.

**Table 1:** Potentially HHT-related diagnoses

Potentially HHT-related diagnoses	Subgroups of diagnoses
Non-traumatic bleedings	CNS bleedings
	GI bleedings
	Epistaxis
	Other unspecified bleedings
	Possible bleedings
Bacterial infections	Infections in joints and bones
	Infections in lower airways
	Infections in wounds and skin
	Other unspecified infections
	CNS infections
	Sepsis
Thromboembolisms	CNS thromboembolisms
	Other unspecified thromboembolisms
Other vascular conditions	Other CNS vascular conditions
	Other unspecified vascular conditions
PAVMs	PAVMs
Other HHT-related contacts	Other HHT-related contacts
Non-HHT-related conditions	Non-HHT-related conditions

### Statistics

Chi-square statistics and Fisher’s exact test were used as appropriate for the analysis of contingency tables.

### Ethics

Permission to extract and analyse data was obtained from the Danish Data Protection Agency (Jnr13/42552) and the Danish National Board of Health (FSEID-00001118).

## Results

All 73 HHT patients and 218 of 219 controls were followed for 20 years. One control was lost to follow-up due to registration failure. During the follow up period, all 73 cases had one or more HHT-relevant hospital visits registered in LPR, whereas 214 of 218 controls had at least one HHT-relevant diagnosis registered. In the manually curated clinical data, only 62 cases and 90 controls were represented with a relevant first event hospital contact (Table 2).

**Table 2:** First event of diagnosis

	LPR data			Clinical data		
	Cases	Controls	*P* value	Cases	Controls	i value
Total number of patients	73	218		73	218	
Patients with at least one relevant event registered	73	214		62	90	
Potentially HHT-related diagnoses						
Non-traumatic bleedings	44 (60%)	42 (19%)	< 0.001	47 (64%)	16 (7%)	< 0.001
Bacterial infections	40 (55%)	84 (38%)	0.015	29 (40%)	61 (28%)	0.06
Thromboembolisms	8 (11%)	38 (17%)	0.19	9 (12%)	29 (13%)	0.83
Other vascular conditions	14 (19%)	48 (22%)	0.610	5 (7%)	32 (15%)	0.0822
PAVMs	6 (8%)	0	< 0.001	27 (37%)	0	< 0.001
Other HHT-related conditions	53 (72%)	0	< 0.001	43 (59%)	0	< 0.001
Non-HHT-related conditions	70 (95%)	210 (96%)	0.8646			
Selected Subgroups of diagnoses						
Epistaxis	27 (37%)	4 (2%)	< 0.001	33 (45%)	2 (1%)	< 0.001
Infections in joints and bones	6 (8%)	1 (0.5%)	< 0.001	5 (7%)	0	< 0.001

The diagnoses were grouped according to clinical relevance for HHT. The time period covers January 1st 1995 to January 1st 2015

The group PAVMs included all patients hospitalised while evaluated for PAVM, in 23 patients PAVM were seen at CT-scan or Pulmonary angiography, while 4 had positive contrast echocardiography, but no PAVM were identified at pulmonary angiography

### Bacterial infections

A significantly increased incidence of infections in joints and bones was identified among cases in both LPR and the clinical dataset (*p*-values < 0.001 respectively, Table 2). In the LPR we identified six cases and one control (five cases and no controls in the clinical dataset, Table 3). None of the cases with infections in joints and bones had in the follow up period been hospitalised due to epistaxis, and four out of six had been examined for PAVMs. Three of these patients were diagnosed with PAVMs and two of them where treated accordingly, the remaining 2 patients declined screening (Table 3). We also found a significantly increased overall incidence of bacterial infections in HHT patients in the LPR data (*P*-value 0.015), but this could not be validated by the clinical data (P-value 0.060.) Based on the clinical data we saw a trend towards lower airway infections being more frequent among cases (P-value 0.051), but this was not evident from the LPR data.

**Table 3:** Infections in Joints and bones and PAVM among cases and controls

	Screening PAVM	PAVM	Age at inclusion	Age at infection	Type of infection LPR	Manual validation
Patient 1	Yes	Small PAVM discovered at time of infection. Embolisation performed	51	69	Osteomyelitis vertebrae	Same
Patient 2	No thanks	?	61	68	Staphylococci, Arthritis and polyarthritis	Same
patient 3	No thanks	?	32	34	Osteomyelitis and periostitis in the mandible and maxillae	False registration, patient excluded from analysis
Patient 4	Yes	No	56	68	Infectious spondylitis purulent intervertebral Discitis	Same
Patient 5	Yes	PAVMs discovered after infection	58	65	Infectious spondylitis or vertebral Osteomyelitis	Same
Patient 6	Yes	Large PAVMs, did not wish to be treated	71	77	Osteomyelitis without specification/ Chronic osteomyelitis,	same
Control 1	Not relevant	–	49	56	Borrelia arthritis	Patient seen in other hospital

Details on all infections in Joints and bones (including type) and presence of PAVM as registered (LPR) in 6 patients and 1 control. In the clinical data patient 3 was excluded as the registration was false. Furthermore, the infection in the control was not identified in the clinical data as the patient was seen in another hospital

### Thromboembolisms and other vascular conditions

In this study, thromboembolic events and other vascular conditions were not more frequent among cases than among controls. Notably, the cases did not present with an increased incidence of thromboembolisms in the CNS (Additional file 2: Table S4, online only.) Though, among the cases that were hospitalised for evaluation of PAVM, 11,1% (3 of 27) had suffered from a CNS thromboembolism. Only 3,7% of the age-and sex-matched controls for this group of cases presented with a thromboembolism in the CNS. This difference was not significant (Fischers exact test *P* value 0.16).

### Non-traumatic bleedings

The percentage of cases with one or more bleeding-related hospitalisation was 60% in the LPR data and 64% in clinical data compared to 19 and 7% for controls, respectively (Table 2). Epistaxis was the most frequent type of bleeding at 37% (LPR data)/45% (clinical data) compared to 0.02% (LPR data)/0.9% (clinical data) among the controls (clinical data) (Table 2). In the clinical data all contacts involving treatment for epistaxis were included. The second most common type of bleeding was “Other bleedings” at 23% (LPR data)/33% (clinical data) compared to 7.3% (LPR data) and 1.8% (clinical data) among the controls (Additional file 2: Table S4, online only). These bleedings were documented bleedings from other organs than GI or epistaxis, or cases where the source of the bleeding was not identified.

The category “Possible bleedings” represented mainly hospitalisations due to anaemia caused by bleeding. This was registered in 31% (LPR data)/ 22% (clinical data) of HHT cases compared to 5.5% (LPR data)/0% (clinical data) of controls (Additional file 2: Table S4, online only). The occurrence of bleeding from the gastrointestinal tract was only documented in 6.8% (LPR data)/14% (clinical data) of cases compared to 1.8% (LPR data)/4.6% (clinical data) of the controls (Table 2). Only spontaneous bleedings were included in the study, and bleedings related to pregnancy/childbirth/abortion or other gynaecologic/obstetric conditions were not included.

### PAVMs

According to the clinical data, 37% (27 cases) were admitted for evaluation of PAVMs in the follow up period. We know from previous data on the same case-group that 31% (23 cases) had PAVM identified on a CT scan, pulmonary angiography or during surgery [23]. Only 8% (6 cases) where registered with a PAVM diagnosis in the LPR.

### Non-HHT-related contacts

The HHT patients and their controls did not differ concerning the initial incidence of non-HHT-related hospitalisations. This was only assessed in the LPR data (Additional file 2: Table S4, online only.)

## Discussion

The present study included patients with HHT from a well-defined geographical area. HHT patients were recruited in a manner that was independent of disease severity through screening of first-degree relatives of known HHT-patients with the aim to reduce referral bias. As expected for an autosomal dominant trait, the patient population had an equal male to female ratio, suggesting that patients had been identified without the introduction of any gender bias. The same investigator performed all the clinical examinations and all individuals fulfilled the clinical diagnostic Curaçao criteria of HHT. The patient population was established prior to the introduction of the Curaçao criteria, however all included individuals fulfilled these at later evaluation. As HHT is typical a misdiagnosed disorder, it is possible that cases with subclinical presentation at the time of investigation may have been missed for inclusion [46]. The fairly high average age of 54 may also affect the outcome, as many of the symptoms of HHT are age-related. The control group was identified among resident of Funen with matching sex and date of birth. The control group was not clinically examined, but in the 20-year follow-up period none of the controls were diagnosed with HHT. Information regarding lifestyle factors such as smoking habits and obesity was not available, which could be a significant confounder, as the HHT cases may have chosen a healthier lifestyle to cope with their condition.

We have analysed the incidence of diagnoses relevant for comorbidity in HHT using first time events. Thereby, we avoid possible bias caused by a few patients with several registrations in the same category.

The diagnoses were obtained in two different ways. Firstly using the LPR registry, which has the advantage that all diagnoses from all hospitals in Denmark are registered here. However, the accuracy of the registered diagnoses is at times limited e.g. when using anaemia as diagnosis, it is often not stated whether it is caused by bleeding. Moreover, it is often not recorded if patients are treated for other conditions as well, when the diagnosis leading to the hospitalisation is determined. Secondly, the diagnoses were obtained by manually examining accessible hospital files, reading the details and changing or adding diagnoses so these would describe the course of a disease in the most accurate manner. The strength of this approach is that several different diagnoses could be captured, and for example the cause of anaemia could be recorded. The limitations are that only diagnoses related to hospitalisations in the county of Funen, were included. It seems that controls have been more inclined to seek residence outside of Funen, based on the presence of only 90 first events out of 218 controls recorded in the clinical data, compared to 214 out of 218 in the LPR data. All cases had a first event according to the LPR data and 62 cases presented with a first event in the clinical data registration. This implies an insufficient access to journal information for controls, and therefore an incomplete registration of relevant events in the clinical data collection, which may underestimate the morbidity of the controls.

### Bacterial infections

It is assumed that HHT patients have an increased risk of severe bacterial infections, and, in particular, the occurrence of cerebral abscesses is increased among HHT-patients with untreated PAVMs [27–29]. The cases included in this study did not present with cerebral abscesses during follow-up. This is expected as a large part of the cases were examined for PAVMs, followed by embolisation of treatable PAVMs. We found indications of increased incidence of infections in general based on the LPR data, but this was not validated in the clinical data. We did, however, detect a significantly increased incidence of infections in joints and bones among the cases compared to controls as previously described [31–35]. These infections are often severe and may require weeks of intravenous antibiotic treatment, underlining the need to prevent their occurrence.

Only few cohort studies have investigated the occurrence of infections among HHT patients. In one of the larger studies [33], it is suggested that HHT is associated with a high frequency of severe infections, defined as any infection that required hospitalisation of the patient. Rather than using a matched control group, they compared their findings to the incidence of sepsis in the United States, and the incidence of *S. aureus* bacteraemia in Europe. Even though the frame of reference for these outcomes may be discussed, the authors found an interesting correlation between median duration of epistaxis, and extra-cerebral infections, leading the authors of the study and those of two case-reports [34, 35] to suggest that nasal colonisation of *S. aureus* and frequent, prolonged epistaxis, generates a gateway for haematogenous dissemination of bacteria to other organs. However, in our study none of our cases with infections in joints and bones had been hospitalised due to epistaxis, which speaks against severe manifestation of this phenotypic event. Unfortunately, any correlation to nasal *S. aureus* colonisation cannot be investigated in the current study design. Furthermore, we did not find any clear correlation between PAVMs and infections in joints and bones as three out of the five cases with these infections (LPR/clinical data) had experienced hospitalisation for evaluation of PAVM, compared to 37% out of all the HHT patients. It is also suggested that HHT patients may have an impaired immune response. This is supported by other studies, linking iron deficiency to alterations in the immune response of HHT patients.This might be part of the explanation for our findings, however further studies are needed in order to address this. Furthermore, increasing age could be a factor inflicting the occurrence of infections in HHT, but as our cases and controls are age matched this cannot explain the difference in this study.

### Thromboembolisms

HHT-patients with untreated PAVMs have an increased risk of ischemic strokes, which is strongly associated with the grade of right-to-left shunting [47]. Additionally, it is found that ischaemic strokes are more common in patients with iron deficiency, suggesting this to be due to enhanced platelet aggregation [48]. The same study suggests that iron deficiency might be an independent risk factor in the general population. Another study finds an association between low serum iron levels, and elevated plasma levels of coagulation factor VIII and venous thromboembolic risk [38]. Elevated plasma levels of FVIII are considered a strong risk factor for VTE in the general population [49]. However, despite being prone to an increased risk of iron deficiency, the cases in our study did not have an increased prevalence of thromboembolic events.

We did not find an increased incidence of ischemic stroke in neither the total HHT patient group nor in the subgroup of HHT patients hospitalised for evaluation of PAVM. In this study cases with PAVMs larger than 2 mm were treated with embolisation in the beginning of the follow-up period. These results imply that treatment of PAVM prevents ischemic stroke by minimising right-to-left shunting. We do not know the conventional stroke risk factors for this cohort, so we need to consider that the cases, to cope with their condition, might have adapted to a healthier lifestyle compared to controls. The careful surveillance and counselling of these patients might also contribute to why this study does not support the theory proposed in the above mentioned papers.

### Non-traumatic bleedings

Bleedings are invalidating for many HHT patients, which is supported by the results of this study. Thus, we found that 64% (clinical data) were treated in the hospital due to bleedings, compared to only 7% of the controls. It is not surprising that epistaxis is the most frequent type of bleeding, and 45% have been treated in the hospital due to this manifestation. In a survey carried out on a US population [50] bleeding-related complications were found to be the most frequent manifestations in hospitalised HHT patients, as they were recorded in 63% of the hospitalised patients. These findings support the results of the current study, stating that bleeding-related manifestations, including anemia, are the most common underlying reason for hospitalisation in HHT-patients. Only 14% of cases (clinical data) were hospitalised due to gastrointestinal bleedings - this is in accordance with the findings of Brinjiki et al. [50]. In a previous study [15] we found that 33% of the current patient population had a history of hematemesis or melena before 1997. This study was based on the patient’s own experience, presented at interview and not restricted to hospitalisation in a specific period of time as in the present data set. The large number of hospitalisations due to anaemia of unknown cause, may partly represent GI bleedings were the source of bleeding was not located.

### PAVMs

In this study, 37% of HHT patients were admitted due to PAVM or PAVM-examination according to the clinical data. As mentioned, we know from previous studies that 32% of the cases in this cohort had PAVMs – the discrepancy between this and our clinical data is caused by a few patients that were admitted for PAVM examination, but where no PAVMs were validated. The prevalence of PAVMs in this study is equivalent to the prevalence suggested in internationally published data and to those found in other studies [23, 51–54]. The low rate of comorbidities for this case group can therefore not be explained by a PAVM prevalence deviation. A pronounced registration bias is evident from the LPR data as only 8% of the cases are here reported hospitalised due to PAVMs. This emphasises the importance of manual validation of data reported to central administrative health registers.

### Other HHT-related contacts and HHT non-related contacts

More than half of the cases had hospital contacts for other reasons than bleedings, infections, thromboembolic events and vascular malformations. These other HHT relevant contacts dealt with information on the HHT disease, examination prior to surgery and questions about HHT’s impact on other health issues. Frequent hospitalisations have a negative impact on quality of life, and therefore further research should investigate how HHT affects the patients’ quality of life.

The cases are not hospitalised more frequently than the controls due to non-HHT-related conditions. This finding validates our diagnoses and diagnosis groups as being potentially HHT relevant or not.

## Conclusion

We found an increased incidence of infections in joints and bones among HHT patients. We consider this important knowledge for clinicians as well as for the patients. Based on the LPR data, HHT patients had an increased overall risk of infections, however this was not validated by the clinical data.

HHT is a rare disease and in spite of the advantages of using a population-based approach, the numbers of patients and outcomes are small in this study. The continuous inclusion of new patients and registration of outcomes in our setting will provide for improved and updated estimates in the future.

Prospective clinical studies are also relevant in order to investigate whether our findings on infections correlate with prolonged epistaxis and nasal *Staphylococcus aureus* colonisation, as has been suggested by others.

We did not find an increased incidence of thromboembolisms, probably because the cases were screened and treated for thrombotic risk factors as PAVMs, anaemia and iron deficiency.

As expected, a large percentage of HHT-patients had hospital contacts due to bleedings compared to the controls, this being the most frequent cause of hospitalisation. Often the exact cause of bleeding was not identified, which likely resulted in an underestimation of the incidence of GI bleedings. The size of this patient cohort was unfortunately too small to examine the extent to which the described comorbidities may vary according to HHT1 and HHT2 genotypes, respectively.

The current study is based on a well-defined population of patients representing various manifestations of HHT. At inclusion, they were enrolled in a specialised HHT Centre with skilled specialists and treatment modalities. This initiative means a thorough follow-up of the patients and treatment of HHT manifestations when necessary. Furthermore, the patients are offered individual information and counselling according to their condition.

Minimising comorbidities is important to optimise the quality of life for patients with HHT. Our findings support that this may be achieved by centralised and intensified treatment.

## Additional files

Additional file 1: **Appendix 1.** Online only, Elaborate description and classification of diagnosis included in the manually collected data. (DOCX 27 kb)

Additional file 2: **Table S4.** Online only, All results. (DOCX 24 kb)

## Abbreviations

ACVRL1: Activin A receptor type II-like 1
AVM: Arteriovenous malformations
CAVM: Cerebral arteriovenous malformation
CNS: Central Nervous System
CT: Computed tomography
ENG: Endoglin
GI: Gastrointestinal
HAVM: Hepatic arteriovenous malformation
HHT: Hereditary Hemorrhagic Telangiectasia
ICD: International Classification of Diseases
JP-HHT: The combined syndrome of Juvenile polyposis and HHT
LPR: Danish abbreviation for Lands Patient Registeret (Danish National Patient Register)
MIM: Mendelian Inheritance in Man
PAVM: Pulmonary arteriovenous malformation
SMAD4: Mothers against decapentaplegic homolog 4

## References

[1] Shovlin CL (2000). Diagnostic criteria for hereditary hemorrhagic telangiectasia (Rendu-Osler-weber syndrome). Am J Med Genet.

[2] Kjeldsen AD, Vase P, Green A (1999). Hereditary haemorrhagic telangiectasia: a population-based study of prevalence and mortality in Danish patients. J Intern Med.

[3] Donaldson JW (2014). The UK prevalence of hereditary haemorrhagic telangiectasia and its association with sex, socioeconomic status and region of residence: a population-based study. Thorax.

[4] Plauchu H (1989). Age-related clinical profile of hereditary hemorrhagic telangiectasia in an epidemiologically recruited population. Am J Med Genet.

[5] Ishikawa T (2010). Pulmonary arteriovenous malformation causing sudden death due to spontaneous hemothorax. Int J Legal Med.

[6] Shovlin CL (1995). Medical complications of pregnancy in hereditary haemorrhagic telangiectasia. QJM.

[7] Donaldson JW (2015). Complications and mortality in hereditary hemorrhagic telangiectasia: a population-based study. Neurology.

[8] Sabba C (2006). Life expectancy in patients with hereditary haemorrhagic telangiectasia. QJM.

[9] de Gussem EM (2016). Life expextancy of parents with hereditary Haemorrhagic telangiectasia. Orphanet J Rare Dis.

[10] Kjeldsen A (2016). 20-year follow-up study of Danish HHT patients-survival and causes of death. Orphanet J Rare Dis.

[11] Gallione CJ (2004). A combined syndrome of juvenile polyposis and hereditary haemorrhagic telangiectasia associated with mutations in MADH4 (SMAD4). Lancet.

[12] Torring PM (2014). National mutation study among Danish patients with hereditary haemorrhagic telangiectasia. Clin Genet.

[13] Folz BJ (2005). Natural history and control of epistaxis in a group of German patients with Rendu-Osler-weber disease. Rhinology.

[14] Hoag JB (2010). An epistaxis severity score for hereditary hemorrhagic telangiectasia. Laryngoscope.

[15] Kjeldsen AD, Kjeldsen J (2000). Gastrointestinal bleeding in patients with hereditary hemorrhagic telangiectasia. Am J Gastroenterol.

[16] Sabba C (2007). Hereditary hemorrhagic telangiectasia: clinical features in ENG and ALK1 mutation carriers. J Thromb Haemost.

[17] Lesca G (2007). Genotype-phenotype correlations in hereditary hemorrhagic telangiectasia: data from the French-Italian HHT network. Genet Med.

[18] Garcia-Tsao G (2000). Liver disease in patients with hereditary hemorrhagic telangiectasia. N Engl J Med.

[19] Fulbright RK (1998). MR of hereditary hemorrhagic telangiectasia: prevalence and spectrum of cerebrovascular malformations. AJNR Am J Neuroradiol.

[20] Haitjema T (1995). Screening family members of patients with hereditary hemorrhagic telangiectasia. Am J Med.

[21] Willemse RB (2000). Bleeding risk of cerebrovascular malformations in hereditary hemorrhagic telangiectasia. J Neurosurg.

[22] Facini C, et al. Hereditary hemorrhagic telangiectasia presenting as migraine: a case report. Brain and Development. 2015. 10.1016/j.braindev.2015.03.004 25857624

[23] Kjeldsen AD (2000). Prevalence of pulmonary arteriovenous malformations (PAVMs) and occurrence of neurological symptoms in patients with hereditary haemorrhagic telangiectasia (HHT). J Intern Med.

[24] Rozenberg D, Leek E, Faughnan ME. Prevalence and nature of dyspnea in patients with hereditary hemorrhagic telangiectasia (HHT). Respir Med. 2015. 10.1016/j.rmed.2015.04.003 25940942

[25] Santhirapala V (2014). Arterial oxygen content is precisely maintained by graded erythrocytotic responses in settings of high/normal serum iron levels, and predicts exercise capacity: an observational study of hypoxaemic patients with pulmonary arteriovenous malformations. PLoS One.

[26] Govani FS, Shovlin CL (2009). Hereditary haemorrhagic telangiectasia: a clinical and scientific review. Eur J Hum Genet.

[27] Kjeldsen AD (2014). Cerebral abscesses among Danish patients with hereditary haemorrhagic telangiectasia. Acta Neurol Scand.

[28] Shovlin CL (2008). Primary determinants of ischaemic stroke/brain abscess risks are independent of severity of pulmonary arteriovenous malformations in hereditary haemorrhagic telangiectasia. Thorax.

[29] Mathis S (2012). Cerebral abscesses in hereditary haemorrhagic telangiectasia: a clinical and microbiological evaluation. Clin Neurol Neurosurg.

[30] Ference BA (1994). Life-threatening pulmonary hemorrhage with pulmonary arteriovenous malformations and hereditary hemorrhagic telangiectasia. Chest.

[31] Servy A (2014). Prognostic value of skin manifestations of infective endocarditis. JAMA Dermatol.

[32] Bally C, Meyssonnier V, Bricaire F (2011). Recurrent Staphylococcus aureus infections. Med Mal Infect.

[33] Dupuis-Girod S (2007). Hemorrhagic hereditary telangiectasia (Rendu-Osler disease) and infectious diseases: an underestimated association. Clin Infect Dis.

[34] Cajander S, Eliasson H (2012). Osler-weber-Rendu syndrome: increased risk of infections and life-threatening complications. Four cases of invasive infectious disease over the same period described. Lakartidningen.

[35] Musso M (2014). Extra-cerebral severe infections associated with haemorrhagic hereditary telangiectasia (Rendu-Osler-weber disease): five cases and a review of the literature. Infez Med.

[36] Guilhem A (2013). Immunological abnormalities associated with hereditary haemorrhagic telangiectasia. J Intern Med.

[37] Cirulli A (2006). Patients with hereditary hemorrhagic Telangectasia (HHT) exhibit a deficit of polymorphonuclear cell and monocyte oxidative burst and phagocytosis: a possible correlation with altered adaptive immune responsiveness in HHT. Curr Pharm Des.

[38] Livesey JA (2012). Low serum iron levels are associated with elevated plasma levels of coagulation factor VIII and pulmonary emboli/deep venous thromboses in replicate cohorts of patients with hereditary haemorrhagic telangiectasia. Thorax.

[39] Clark K, Pyeritz RE, Trerotola SO (2013). Angina pectoris or myocardial infarctions, pulmonary arteriovenous malformations, hereditary hemorrhagic telangiectasia, and paradoxical emboli. Am J Cardiol.

[40] Wu W (2014). Paradoxical coronary embolisms as a presentation of hereditary hemorrhagic telangiectasia. J Am Coll Cardiol.

[41] Janion M (2010). Myocardial infarction in a low risk patient with hereditary hemorrhagic telangiectasia. Cardiol J.

[42] Kjeldsen AD (2005). Clinical symptoms according to genotype amongst patients with hereditary haemorrhagic telangiectasia. J Intern Med.

[43] Kjeldsen, A.D., Epidemiological and clinical aspects of Hereditary Haemorrhagic Telangiectasia, Mb Osler, in Otorhinolaryngology. 1998, University of Southern Denmark.

[44] Andersen PE, Kjeldsen AD (2010). Interventional treatment of pulmonary arteriovenous malformations. World J Radiol.

[45] Lynge E, Sandegaard JL, Rebolj M (2011). The Danish National Patient Register. Scand J Public Health.

[46] Sabba C (2005). A rare and misdiagnosed bleeding disorder: hereditary hemorrhagic telangiectasia. J Thromb Haemost.

[47] Velthuis S (2013). Grade of pulmonary right-to-left shunt on contrast echocardiography and cerebral complications: a striking association. Chest.

[48] Shovlin CL (2014). Ischaemic strokes in patients with pulmonary arteriovenous malformations and hereditary hemorrhagic telangiectasia: associations with iron deficiency and platelets. PLoS One.

[49] Kraaijenhagen RA (2000). High plasma concentration of factor VIIIc is a major risk factor for venous thromboembolism. Thromb Haemost.

[50] Brinjikji W (2016). High rates of bleeding complications among hospitalized patients with hereditary hemorrhagic telangiectasia in the United States. Ann Am Thorac Soc.

[51] Begbie ME, Wallace GM, Shovlin CL (2003). Hereditary haemorrhagic telangiectasia (Osler-weber-Rendu syndrome): a view from the 21st century. Postgrad Med J.

[52] Ni Bhuachalla CF (2010). Experience of the Irish National Centre for hereditary haemorrhagic telangiectasia 2003-2008. Respir Med.

[53] Al-Saleh S (2009). Screening for pulmonary and cerebral arteriovenous malformations in children with hereditary haemorrhagic telangiectasia. Eur Respir J.

[54] Giordano, P., et al., Hereditary hemorrhagic telangiectasia: arteriovenous malformations in children*.* J Pediatr, 2013. 163(1): p. 179–186.e1–3. 10.1016/j.jpeds.2013.02.009 23535011

