# Supplementary material for: Comorbidity among HHT patients and their controls in a 20 years follow-up period
Source: Orphanet J Rare Dis. 2018 Dec 14;13:223. doi: 10.1186/s13023-018-0962-8 (PMC6295040; doi:10.1186/s13023-018-0962-8)
Supplement: Supplementary file 1 — Appendix 1. Online only, Elaborate description and classification of diagnosis included in the manually collected data. (DOCX 27 kb) [file 13023_2018_962_MOESM1_ESM.docx]

**Appendix 1. Elaborate description and classification of diagnosis included in the clinical data**

General criteria regarding data included from clinical charts:

- Only contacts leading hospitalisation are included, apart from contacts regarding “Epistaxis”, and “Other HHT-related contacts” (the latter two only accounted for in the clinical data), Inclusion of contacts is based on the primary discharge diagnosis only.
- From the clinical data, secondary discharge diagnoses are included if the condition from a clinical view would have led to hospitalisation nonetheless.
- The date for included diagnoses = the first day of the hospitalisation period in to
- question.
- Regarding outpatient visits included in the clinical data collection, every physical visit to the hospital is accounted for.
- Conditions related to pregnancy/childbirth/abortion/breastfeeding or other gynecologic/obstetric/ neonatal/congenital conditions are not included.
- Only contacts represented by a journal or notes are included in the clinical data. Contacts referred to, but not registered, are therefore not included.
- In the clinical data, hospital admissions in which patients are transferred to another ward or hospital are considered as one hospitalisation if it is not a new condition that leads to the transfer.
- For all contacts included in the clinical data, the ICD10 classification is evaluated according to the journal information available, and if these are not consistent, the most appropriate diagnosis code is chosen for the contact in question.
- Bacterial infections
- Conditions primarily caused by bacteria are included. Therefore, inflammations or infections primarily caused by other determinants are left out.
- Bacterial infections rarely seen in Denmark are left out.
- Infections that are predominantly sexually transmitted, are not included.
- Ulcers in the gastrointestinal tract are considered an infection, but if bleeding complications has occurred, these contacts are categorised as “Bleedings in the gastrointestinal tract”.
- Hospital admissions due to postsurgical infections are included. For the clinical data, B-diagnoses within this category are also included when leading to re-surgery or i.v. antibiotic treatment.
- Infections coded as B-diagnoses are included if treated with i.v. antibiotics, or if it from a clinical view would have led to hospitalisation nonetheless.

Thromboembolisms

- Phlebitis is not considered a thromboembolic condition, but an inflammation and therefore not included.
- Hospitalisations caused by sequela due to thromboembolisms are also included in this category.

Non-traumatic bleedings

- Only spontaneous bleedings are included. Therefore, surgical related bleedings or bleeding related to other procedures or trauma, are not included.
- Bleedings as a symptom to cancers, polyps or other conditions, are only included in the clinical data, if the bleeding itself causes the hospitalisation.
- Anaemia diagnoses that could have been due to bleeding are included as “possible bleedings”. In the clinical data, admissions due to these diagnoses are carefully evaluated, and if it is noted that bleeding is not considered the reason for anaemia; if another reason is considered the most likely; or if no reasoning has been done at all, the contact is not included. If it on the contrary is determined that the anaemia is caused by bleeding, the ICD10 classification is altered according to relevance.

Other vascular conditions

- Aneurisms, vascular fistulas and arteriosclerotic conditions without thrombosis are included.
- Vasculitis, other vascular inflammations and congenital vascular conditions are not included.
- Admissions due to not specified heart failure or non-arteriosclerotic valve diseases are not included. They are included in the clinical data if arteriosclerosis is described to having caused the condition. The ICD10 classification is then altered according to relevance.

Pulmonary arterio-venous malformations (PAVMs)

- Contacts due to screening for PAVMs with echocardiography or pulmonary CT scanning are not included.
- Contacts due to pulmonary angiography with or without embolisation are included. The majorities of these had visible PAVMs, But the small subgroup of patients who had positive contrast echocardiography but no PAVM at Pulmonary angiography were also included in this group

Epistaxis

- Both outpatient visits and hospital admissions are included. For the clinical data, the contacts are included if the patient has received any kind of treatment for epistaxis. A contact caused by epistaxis in which no treatment is given, the contact is included in the category “other HHT-related contacts”.

Other HHT-related contacts

- These data only regard HHT patients.
- Both outpatient visits and hospital admissions are included.
- In the data extracted from databases, this regards all contacts with the diagnosis code DI780 or DI780A.
- In the clinical data, this includes all hospital contacts caused by HHT, that are not included in any other HHT relevant category and where the patient is seen by a doctor or receives treatment. This does not involve contacts related to participation in research projects, or laboratory tests.
- Medical assistance regarding HHT during admission to hospital (regarding another issue), are included as a contact if it is estimated that the condition would have led to hospital contact nonetheless.
- Contacts related to pulmonic hypertension are included, as this directly correlates with HHT.
- Contacts regarding conditions that a suspected to be caused by HHT are only included if the causality is proven.
- Contacts registered under the diagnoses DI780/DI780A (HHT) are in the clinical data - when possible - given a diagnosis code more accurate for the specific contact. Frequent alterations in this context are hospitalisations due to none specified bleeding coded DD500, hospitalisations because of PAVMs coded DI280, contacts due to epistaxis coded DR040.

Non-HHT-related conditions

- Only assessed in the LPR data and include all hospitalisations that are not categorised as HHT related.
