# Supplementary material for: Comorbidity among HHT patients and their controls in a 20 years follow-up period
Source: Orphanet J Rare Dis. 2018 Dec 14;13:223. doi: 10.1186/s13023-018-0962-8 (PMC6295040; doi:10.1186/s13023-018-0962-8)
Supplement: Supplementary file 2 — Table S4. Online only, All results. (DOCX 24 kb) [file 13023_2018_962_MOESM2_ESM.docx]

**Table S4. Online only. All results**

|  | LPR* | | | Clinical data | | |  |
| --- | --- | --- | --- | --- | --- | --- | --- |
|  | **Cases** | **Controls** | **P-value** | **Cases** | **Controls** | **P-value** |  |
| Total number of patients | 73 | 218 |  | 73 | 218 |  |  |
| Patients with at least one relevant event registered | 73 (100%) | 214 (98%) |  | 62 (84%) | 90 (41%) |  |  |
| Potentially HHT-related diagnoses |  | | | | | |  |
| Non-traumatic bleedings | 44 (60%) | 42 (19%) | <0.001 | 47 (64%) | 16 (7%) | <0.001 |  |
| Bacterial infections | 40 (55%) | 84 (38%) | 0.015 | 29 (40%) | 61 (28%) | 0.060 |  |
| Tromboembolisms | 8 (11%) | 38 (17%) | 0.190 | 9 (12%) | 29 (13%) | 0.831 |  |
| Other vascular conditions | 14 (19%) | 48 (22%) | 0.608 | 5 (7%) | 32 (15%) | 0.082 |  |
| PAVMs | 6 (8%) | 0 | <0.001 | 27 (37%) | 0 | <0.001 |  |
| Other HHT-related contacts | 53 (72%) | 0 | <0.001 | 43 (59%) | 0 | <0.001 |  |
| Non-HHT-related conditions | 70 (95%) | 210 (96%) | 0.865 |  | | |  |
| Subgroups of diagnoses |  | | | | | |  |
| CNS bleedings | 5 (7%) | 4 (2%) | 0.032 | 1 (1%) | 1 (0.5%) | 0.049 | |
| GI bleedings | 5 (7%) | 4 (2%) | 0.032 | 10 (23%) | 10 (9%) | 0.008 | |
| Epistaxis | 27 (37%) | 4 (2%) | <0.001 | 33 (45%) | 2 (1%) | <0.001 | |
| Other unspecified bleedings | 17 (23%) | 16 (7%) | <0.001 | 24 (33%) | 4 (2%) | <0.001 | |
| Possible bleedings | 23 (32%) | 12 (6%) | <0.001 | 16 (22%) | 0 | <0.001 | |
| Infections in joints and bones | 6 (8%) | 1 (0.5%) | <0.001 | 5 (7%) | 0 | <0.001 | |
| Infections in lower airways | 14 (19%) | 32 (15%) | 0.362 | 15 (21%) | 25 (12%) | 0.051 | |
| Infections in wounds and skin | 7 10%) | 16 (7%) | 0.538 | 2 (3%) | 14 (6%) | 0.232 | |
| Other unspecified infections | 22 (30%) | 60 (28%) | 0.667 | 14 (19%) | 38 (17%) | 0.736 | |
| CNS infections | 0 | 0 |  | 0 | 0 |  | |
| Sepsis | 4 (6%) | 8 (4%) | 0.501 | 4 (6%) | 7 (3%) | 0.379 | |
| CNS Thromboembolisms | 6 (8%) | 21 (10%) | 0.719 | 5 (7%) | 17 (8%) | 0.791 | |
| Other unspecified tromboembolisms | 3 (4%) | 23 (11%) | 0.095 | 5 (7%) | 16 (7%) | 0.889 | |
| Other CNS vascular conditions | 3 (4%) | 11 (5%) | 0.746 | 2 (3%) | 8 (4%) | 0.706 | |
| Other unspecified vascular conditions | 11 (15%) | 41 (19%) | 0.471 | 3 (4%) | 26 (12%) | 0.054 | |

* LPR: Landspatientregisteret (Danish National Patient Register)

First event of a diagnosis including all subgroups. The diagnoses were grouped according to clinical relevance for HHT. The time period covers 1.1.1995-1.1.2015 The group PAVMs included all patients hospitalised while evaluated for PAVM, in 23 patients PAVM were seen at CT-scan or Pulmonary angiography, while 4 had positive contrast echocardiography, but no PAVM were identified at pulmonary angiography.
